# HPLC-MS and Network Pharmacology Analysis to Reveal Quality Markers of Huo-Xue-Jiang-Tang Yin, a Chinese Herbal Medicine for Type 2 Diabetes Mellitus

**DOI:** 10.1155/2021/1072975

**Published:** 2021-03-15

**Authors:** Qiugu Chen, Yuan Zhao, Maosheng Li, Ping Zheng, Shangbin Zhang, Huilin Li, Jianping Chen

**Affiliations:** ^1^Shenzhen Key Laboratory of Hospital Chinese Medicine Preparation, Shenzhen Traditional Chinese Medicine Hospital, Shenzhen 518033, China; ^2^The Fourth Clinical Medical College of Guangzhou University of Chinese Medicine, Shenzhen 518033, China; ^3^Department of Endocrinology, Shenzhen Traditional Chinese Medicine Hospital, Shenzhen 518033, China

## Abstract

Huo-Xue-Jiang-Tang Yin (HXJTY) is a Chinese medicine formulation, which has been widely used for the treatment of various lipometabolism- and glycometabolism-related diseases in clinics. Currently, HXJTY is mainly prescribed to treat patients with type 2 diabetes mellitus (T2DM), yet its chemical and pharmacologic profiles remain to be elucidated. Here, the potential bioactive compound and action mechanism were investigated using chemical and network pharmacology analysis. A rapid HPLC-MS was employed to identify and quantify the component of HXJTY. On the basis of the identified chemical markers from HXJTY, a network pharmacology study, including target gene prediction and functional enrichment, was applied to screen out the main quality markers of HXJTY and explore its potential mechanism for the treatment of T2DM. The results showed that a total of 22 components were identified and quantified from HXJTY by HPLC-MS. Furthermore, 12 active components such as astragaloside IV, calycosin-7-O-*β*-D-glucoside, hydroxysafflor yellow A, and others were proposed as quality markers of HXJTY for treating T2DM based on network pharmacology analysis. In addition, 125 corresponding possible therapeutic target genes of T2DM were obtained. These target genes are mainly related to peptidase activity, hydrolase activity, phosphatase activity, and cofactor binding, suggesting the involvement of PI3K-Akt, MAPK, AGE-RAGE, and Rap1 signaling pathways in HXJTY-treated T2DM. Our results may provide a useful approach to identify potential quality markers and molecular mechanism of HXJTY for treating T2DM.

## 1. Introduction

Huo-Xue-Jiang-Tang Yin (HXJTY), a Chinese herbal decoction, has been widely used for the treatment of type 2 diabetes mellitus (T2DM). HXJTY is composed of eight different herbs, that is, Astragali Radix (roots of *Astragalus membranaceus* (Fisch.) Bge. var. *mongholicus* (Bge.) Hsiao), Rehmanniae Radix (root tubers of Rehmannia glutinosa Libosch.), Carthami Flos (flowers of Carthamus tinctorius L.), Ophiopogonis Radix (root tubers of Ophiopogon japonicus (L.f) Ker-Gawl.), Rhei Radix Et Rhizoma (roots and rhizomes of *Rheum palmatum* L.), Persicae Semen (seeds of *Prunus persica* (L.) Batsch), Dioscoreae Rhizoma (rhizomes of *Dioscorea opposita* Thunb.), and Pseudostellariae Radix (root tubers of *Pseudostellaria heterophylla* (Miq.) Pax ex Pax et Hoffm.). Recently, patients may choose alternative therapeutic options, such as traditional Chinese medicine (TCM), to improve quality of life in diabetes. HXJTY has also been commonly prescribed to patients with T2DM in clinics [1, 2]. However, due to complicated chemical constituents, the quality markers and specific molecular mechanism of HXJTY for treating T2DM are still not clear. The quality control of TCM is currently a great concern, and when it is not available, its effect is often questionable. It is therefore necessary to investigate the potential bioactive markers and mechanism of action of HXJTY.

Generally, most quality control approaches of TCM preparations are detected by chromatographic methods, such as HPLC, UPLC, and MS [3, 4]. Although these traditional analytical methods can be used to analyze the various components of TCM, concerns about the active components or quality markers of TCM continue to be raised. Distinguishing the active components, which are consistently associated with efficacy, from the chemical markers has therefore attracted more attention in relation to quality control of TCM. In 2007, Hopkins proposed a new conception of network pharmacology [5], offering a perfect paradigm to deal with multitarget combination drugs. Network pharmacology has recently been successfully adopted to investigate the potential active ingredients for quality control of numerous TCM formulations [6–8].

**Figure 1:**
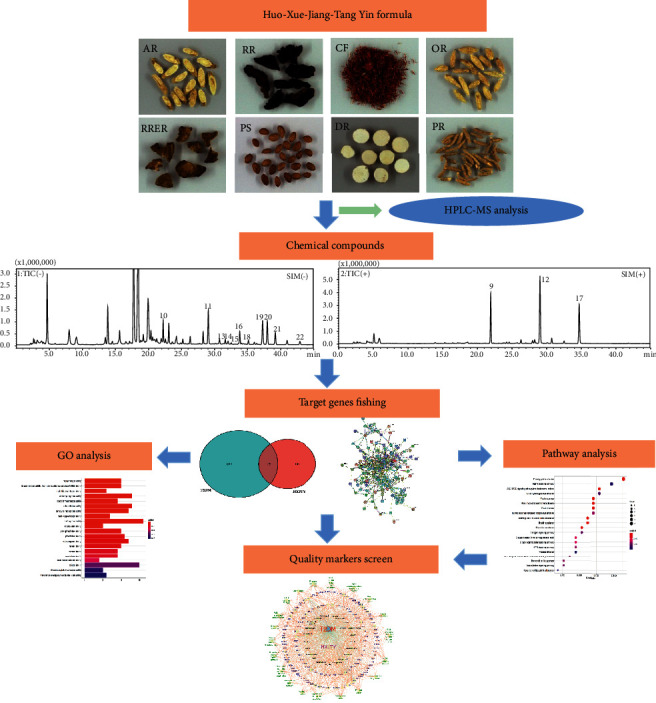
The workflow for the network pharmacology approach used in our study.

At present, application of network pharmacology to explore TCM in terms of target gene identification and function prediction is mostly based on the chemical components derived from each herb of TCM in the existing database. Since TCM preparation goes through extraction, purification, and other processes, its chemical composition may exhibit variation compared with that of ingredients in the original medicinal material. Therefore, active components that can be identified and quantified within the final product of TCM preparation should be chosen as the appropriate and accurate quality markers for quality control indication. In this study, we aim to identify quality markers for quality control indication of HXJTY so as to ensure the safety and efficacy of HXJTY. Here, the components in HXJTY will be identified and quantified by HPLC-MS, and then the quality markers of HXJTY for treating T2DM are further selected from the detected components by network pharmacology. Based on the findings, potential quality markers and the mechanism of action of HXJTY for treating T2DM will be revealed. The design of this experiment is shown in Figure 1.

## 2. Materials and Methods

### 2.1. Chemicals and Reagents

Catalpol (1), aucubin (2), gallic acid (3), hydroxysafflor yellow A (6), amygdalin (7), echinacoside (8), calycosin-7-O-*β*-D-glucoside (9), acteoside (10), astragaloside IV (13), rhein (19), and emodin (22) were purchased from China Institute of Food and Drug Verification and Research (Beijing, China). Rhmannioside D (4), leonuride (5), ononin (11), calycosin (12), astragaloside III (14), ophiopojaponin C (15), astragaloside II (16), formononetin (17), isoastragaloside II (18), astragaloside I (20), and isoastragaloside I (21) were purchased from Sichuan Victory Biological Technology Co., Ltd. (Chengdu, China). Chromatographic grade acetonitrile and formic acid were purchased from Merck (Darmstadt, Germany). Deionized water was purified using a Milli-Q system (Aquapro, USA).

### 2.2. Preparation of Sample Solution

Eight different herbs such as Astragali Radix were used according to the prescribed proportions and were extracted in boiling water (3 L) twice. After filtration, the extract was dried using a freeze-drying machine, and it was stored in a refrigerator at −80°C before injection into HPLC system for analysis.

0.4 g powder of the HXJTY was accurately weighed and sonicated in 10 mL of 50% methanol by ultrasonic extraction for 30 min at 25°C. The weight loss was compensated by adding 50% methanol after extraction. Then, the solution was centrifuged at 4,500 rpm for 10 min, and the supernatant was filtered through a membrane with 0.45 *µ*m pores for analysis. All solutions were stored at 4°C until use.

### 2.3. HPLC Chromatography and MS Conditions

Liquid chromatographic separation and mass spectrometric detection were performed using Shimadzu LC-20AT HPLC system coupled with a tandem dipole mass spectrometer (Shimadzu, Japan). The instrument was equipped with ESI sources. Chromatographic separation was accomplished on Waters XSelect HSS T3 (250 mm × 4.6 mm, 5 *μ*m) at a flow rate of 1.0 mL/min. The column temperature was maintained at 30°C. The mobile phase was composed of acetonitrile (A) and water containing 0.1% formic acid (B) with the following gradient elution program: 0–5 min, 5%A; 5–45 min, 5%A ⟶ 78%A. The injection amount was 10 *μ*L. The MS spectra were acquired with SIM mode in both positive and negative ion modes. The MS conditions were optimized as follows: detector voltage, 3.5 kV; atomizer pressure, 241 kPa; ion spray temperature, 450°C; dry gas flow, 1.5 L/min.

### 2.4. Prediction of Potential Target Gene for the Quantitative Component and Disease

All candidate target genes of the chemical component quantified by HPLC-MS were predicted using Integrative Pharmacology-based Research Platform of Traditional Chinese Medicine (TCMIP) v2.0 (http://www.tcmip.cn/) and Swiss Target Prediction (http://www.swisstargetprediction.ch/). The analysis platform was mainly based on the structural similarity evaluation with a known ingredient to predict the potential target gene of the objective component. In this study, a Tanimoto score with more than 0.80 was chosen as a potential target gene of the chemical component. The target gene of disease was predicted using OMIM (https://www.omim.org/) and GeneCards (https://www.genecards.org/) platform with type 2 diabetes mellitus as the keyword.

### 2.5. Screening of the Common Target Genes and Mutual Network Construction for the Quantitative Component and the Disease

The common target genes of the quantitative component and the disease were obtained using the *R* programming language (https://www.r-project.org/), and a Venn diagram was drawn. Protein–protein interaction (PPI) network of the common target genes was constructed by STRING platform, and the minimum interaction threshold was set to “highest confidence” (>0.9). Meanwhile, a histogram was drawn to reflect statistical frequency of the top 30 common target genes.

### 2.6. Gene Ontology (GO) Analysis and Kyoto Encyclopedia of Genes and Genomes (KEGG) Pathway Enrichment Analysis

GO analysis and KEGG pathway enrichment analysis of the common target genes was done using the *R* programming language software, and the results were presented as a bar chart and bubble chart, respectively.

### 2.7. Compound Prescription-Active Component-Disease-Target Gene-Pathway Interaction Network Analysis

Compound prescription-active component-disease-target gene-pathway interaction network was constructed by Cytoscape 3.71 software. In the network diagram, “node” represented the drug, active component, disease, target gene, and pathway, respectively. “Edge” represented the relationship between the aforementioned nodes. The topological attributes of the aforementioned results were analyzed and filtered by network analysis plug-in of Cytoscape software. The quality markers were evaluated by degree; all of the active components greater than the average of degree in the network were selected for screening. Degree is the most direct method to characterize the centrality of node in the network analysis [9].

## 3. Results

### 3.1. Quantification of Chemical Components of HXJTY by HPLC-MS

**Figure 2:**
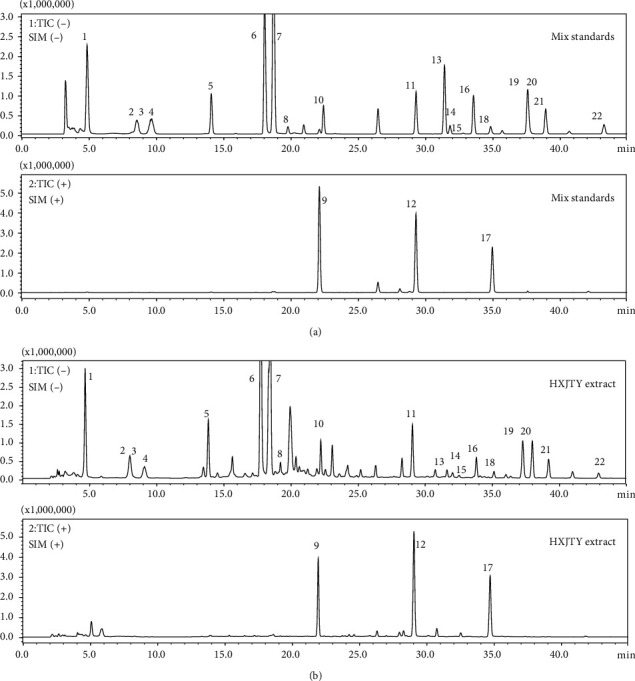
The HPLC-MS analysis SIM chromatogram of 22 analytes. (a) Mix standards; (b) HXJTY extract; (1) catalpol; (2) aucubin; (3) gallic acid; (4) rhmannioside D; (5) leonuride; (6) hydroxysafflor yellow A; (7) amygdalin; (8) echinacoside; (9) calycosin-7-O-*β*-D-glucoside; (10) acteoside; (11) ononin; (12) calycosin; (13) astragaloside IV; (14) astragaloside III; (15) ophiopojaponin C; (16) astragaloside II; (17) formononetin; (18) isoastragaloside II; (19) rhein; (20) astragaloside I; (21) isoastragaloside I; (22) emodin.

**Figure 3:**
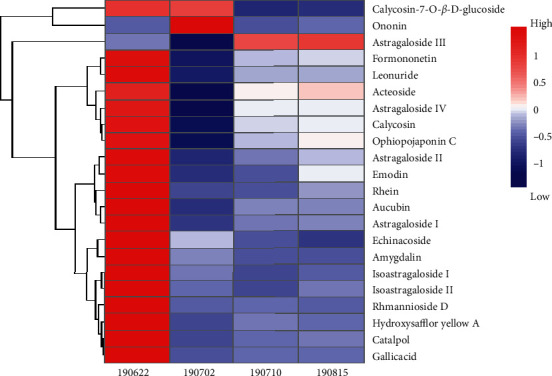
Thermograms of the content of 22 components. Data are expressed as an average of five independent samples (*n* = 5); the color from red to blue represents the concentration from high to low.

In order to achieve comprehensive quality control of HXJTY water extract, an HPLC-MS approach was established to simultaneously quantify 22 components in HXJTY in this study. The monitoring ion for detection and analysis is shown in Table 1 and Figure 2. The results of the detected components are summarized in Figure 3. Here, a total of 22 identified chemical markers were listed as candidate compounds for further network pharmacology analysis.

### 3.2. Target Gene Screening and Interaction Network Construction

**Figure 4:**
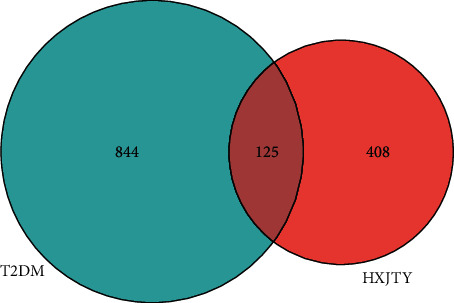
Venn diagram of the common target genes for compound prescription and disease. The circle's size represents the number of the target genes, the blue circle represents the target genes of T2DM, the red circle represents the target genes of 22 quantitative components in HXJTY, and the coincident part represents the common target genes.

**Figure 5:**
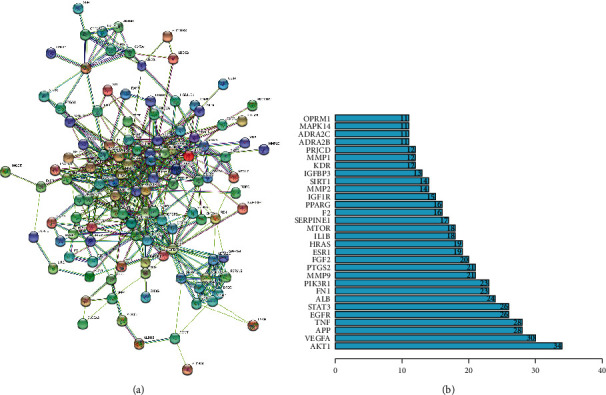
The result of the common target genes network interaction. (a) PPI network of the common target genes. The nodes represent target genes; the stuffing of the nodes represent 3D structure of target genes; the edges represent target genes-target genes associations; the colors of the edges represent different interactions; cyan and purple represent known interactions; green, red, and blue purple represent predicted interactions; chartreuse, black, and light blue represent others. (b) Frequency of the top 30 common target genes.

As shown in Figure 4, for the Venn diagram, a total of 533 potential target genes were obtained for the 22 quantitative components of HXJTY. Meanwhile, 969 disease target genes associated with T2DM were retrieved using OMIM and the GeneCards platforms. One hundred twenty-five shared common target genes were identified between the quantitative components of HXJTY and T2DM by using the R programming language analysis. Among 22 components, acteoside was found to have no common target gene with T2DM. Therefore, the remaining 21 components in HXJTY including catalpol, aucubin, gallic acid, rhmannioside D, leonuride, hydroxysafflor yellow A, amygdalin, echinacoside, calycosin-7-O-*β*-D-glucoside, ononin, calycosin, astragaloside IV, astragaloside III, ophiopojaponin C, astragaloside II, formononetin, isoastragaloside II, rhein, astragaloside I, isoastragaloside I, and emodin were targeted for further analysis. The common target genes PPI diagram indicated that there were 115 nodes and 972 edges in PPI (Figure 5(a)). The frequency of occurrence of the top 30 common target genes was shown in Figure 5(b). AKT1, VEGFA, APP, TNF, EGFR, STAT3, and other target genes exhibited high frequency of protein interaction, which may be the node protein of the whole network. The results showed that the selected components of HXJTY had a high binding activity with them and could be used as the potential target genes of HXJTY for treating T2DM.

### 3.3. Screening of Key Pathways of HXJTY for Treating T2DM

**Figure 6:**
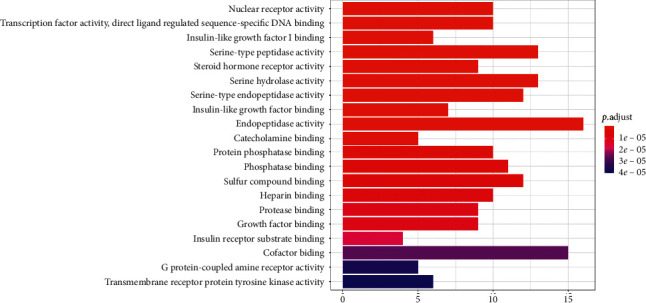
GO biological process analysis (top 20). The node length represents the number of target genes enriched, and the node color from blue to red represents the *P* value from large to small.

**Figure 7:**
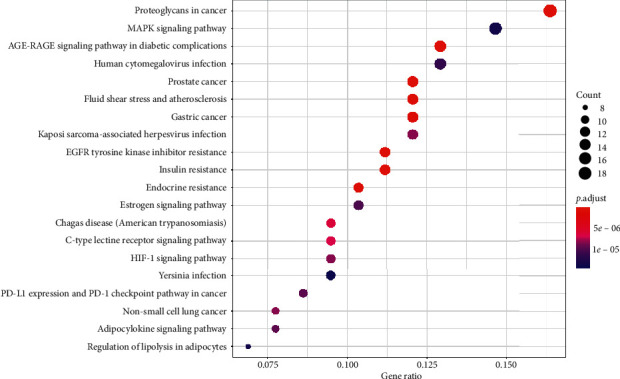
KEGG pathway enrichment analysis (top 20). The node size represents the number of target genes enriched, and the node color from blue to red represents the *P* value from large to small.

GO analysis of the common target genes showed that the biological process was mainly involved in peptidase activity, hydrolase activity, phosphatase activity, and cofactor binding (Figure 6). KEGG pathway enrichment analysis of the aforementioned common target genes using *R* language software is presented in Figure 7. After exclusion of broad pathways, the top 20 signaling pathways are listed in Table 2, among which 125 common target genes were mainly related to PI3K-Akt, MAPK, AGE-RAGE, Rap1, and other multiple signaling pathways. This suggested that the effect of HXJTY for treating T2DM may act on multiple pathways, as well as complex interactions among these pathways.

### 3.4. Compound Prescription-Active Component-Disease-Target Gene-Pathway Interaction Network

**Figure 8:**
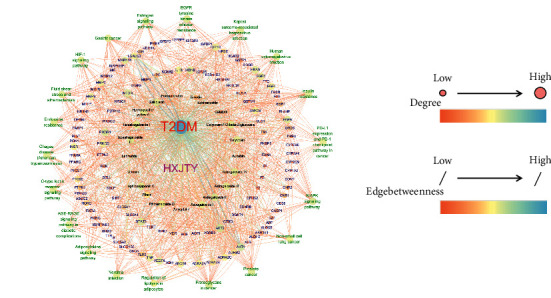
Compound prescription-active component-disease-target gene-pathway interaction network. The word colors represent different groups: red represents the disease; pink represents the compound prescription; black represents the active component; blue represents the target gene; green represents the pathway; node size and color represent the degree; the degree from low to high represents the node from small to large and the color from orange to cyan; edge size and color represent edgebetweenness; edgebetweenness from low to high represents the edge from thin to thick and the color from orange to cyan.

Compound prescription-active component-disease-target gene-pathway interaction network finding is shown in Figure 8. The network contained a total of 168 nodes (125 target genes, 21 active components, 1 disease, 1 compound prescription, and 20 KEGG pathways (top 20)) and 1362 edges. Besides, the interaction network results of 21 active compounds are shown in Table 3. The degree of catalpol, aucubin, gallic acid, rhmannioside D, leonuride, hydroxysafflor yellow A, amygdalin, echinacoside, calycosin-7-O-*β*-D-glucoside, ononin, calycosin, astragaloside IV, astragaloside III, ophiopojaponin C, astragaloside II, formononetin, isoastragaloside II, rhein, astragaloside I, isoastragaloside I, and emodin were 1, 1, 27, 17, 1, 24, 1, 2, 31, 3, 27, 31, 26, 28, 31, 4, 32, 11, 27, 28, and 8, respectively. The average value of 168 nodes in the network is 12. Thus, gallic acid, rhmannioside D, hydroxysafflor yellow A, calycosin-7-O-*β*-D-glucoside, calycosin, astragaloside IV, astragaloside III, ophiopojaponin C, astragaloside II, isoastragaloside II, astragaloside I, and isoastragaloside I can be selected as quality markers of HXJTY for treating T2DM. The aforementioned results show that quality markers in HXJTY may act on the whole biological network system, rather than acting on a single target gene. It embodied the integrity and complexity of the function of TCM and also explained the mechanism of HXJTY for treating T2DM with multicomponent, multitarget, and multipathway.

## 4. Discussion

The characteristic of TCM prescription is multicomponent, multitarget, and multiapproach, such that the single component cannot reflect the actual curative effect of TCM. Therefore, it is of a great challenge to find an appropriate approach to control the quality of TCM decoctions and their products. One of the TCM preparations, HXJTY, has been commonly used to treat patients with T2DM, but a suitable quality control method is not available to assure the efficacy of HXJTY. Recently, HPLC combined with MS has been widely employed to establish a quality control method by qualitative and quantitative determination of the major constitutes within herbal medicine [10, 11]. However, it is still not able to distinguish the quality markers, which represent the efficacy to the greatest extent in the whole prescription, from a large number of chemical markers. Network pharmacology provides an ideal paradigm to deal with multitarget combination drugs and has recently been successfully employed to predict the active ingredients in TCM formulae [7]. In this study, we thus combined HPLC-MS chemical profile with network pharmacology to predict potential bioactive compound to elucidate the potential quality markers of HXJTY.

First, in the HPLC-MS chemical analysis, we simultaneously tested 22 chemical components in HXJTY, including 10 constituents (saponins and flavonoids) in Astragali Radix, eight constituents (iridoids glycosides) in Rehmanniae Radix, three constituents (anthraquinones) in Rhei Radix et Rhizoma, one constituent (flavonoids) in Carthami Flos, one constituent (saponins) in Ophiopogonis Radix, and one constituent (glycosides) in Persicae Semen. Second, 21 of them were selected as active components by target gene screening of network pharmacology. Finally, according to the degree in compound prescription-active component-disease-target gene-pathway interaction network, gallic acid, rhmannioside D, hydroxysafflor yellow A, calycosin-7-O-*β*-D-glucoside, calycosin, astragaloside IV, astragaloside III, ophiopojaponin C, astragaloside II, isoastragaloside II, astragaloside I, and isoastragaloside I were selected as quality markers of HXJTY for treating T2DM. Modern pharmacological studies have confirmed that saponins and flavonoids have significant physiological and pharmacological activities and also exhibit beneficial effect in the prevention and treatment of T2DM [12, 13]. In support of this, flavonoids and saponins from Astragali Radix have been reported to possess the ability to prevent T2DM [14, 15]. For instance, astragaloside IV increased basal and insulin-stimulated glucose uptake in C2C12 myotubes and decreased the mRNA expression of inflammatory cytokines through suppression of the IKK/I*κ*B*α* pathway activation in insulin resistance model of C2C12 myotubes [16]. Besides, astragaloside IV also showed potential therapeutic effects on diabetic nephropathy, diabetic vascular disease, diabetic retina, and other diabetic complications [17]. Tang et al. also discovered that calycosin-7-O-*β*-D-glucoside and calycosin, two major flavonoids within Astragali Radix, inhibited high glucose-induced mesangial cell early proliferation and AGEs-mediated cell apoptosis in glomerular endothelial cells [18]. The results indicate that both calycosin-7-O-*β*-D-glucoside and calycosin had a significant therapeutic potential in modulating the progression and/or development of diabetic nephropathy. Hydroxysafflor yellow A, one of the major water-soluble and bioactive components in Carthami Flos, has been found to be effective in chronic metabolic diseases such as T2DM and obesity [19, 20]. Adipocytes play a vital role in regulating adipose mass, T2DM, and obesity [21]. Zhu et al. showed that hydroxysafflor yellow A restrained the proliferation of 3T3-L1 preadipocytes and reduced the lipid accumulation in 3T3-L1 cells [22]. A similar result was obtained by Yu et al. who demonstrated that the triglyceride levels of adipocytes in bone marrow stromal cells treated with hydroxysafflor yellow A were significantly lower than that of control group [23]. The aforesaid findings suggest that hydroxysafflor yellow A can suppress the content of triglycerides in adipocytes by promoting the lipolysis and may further have a beneficial role in T2DM.

According to TCM theory, both Astragali Radix and Rehmanniae Radix possessing primary pharmacological action against the main cause of a disease are considered as monarch drugs in the prescription. Carthami Flos and Ophiopogonis Radix with the ability to treat cardinal or concurrent symptoms are proposed as minister drugs in the prescription [24]. From the results of network pharmacological screening, 12 kinds of quality markers are all contributed by monarch and minister drugs, in which calycosin-7-O-*β*-D-glucoside, calycosin, astragaloside IV, astragaloside III, astragaloside I, isoastragaloside I, astragaloside II, and isoastragaloside II are derived from Astragali Radix, gallic acid and rhmannioside D are derived from Rehmanniae Radix, hydroxysafflor yellow A is derived from Carthami Flos, and ophiopojaponin C is derived from Ophiopogonis Radix. Therefore, the current findings also further supported the organizing principle of “monarch-minister-adjuvant-conductant” in TCM practitioners. It can be seen from Table 1 that the bioactive components of HXJTY in treating T2DM are mainly glycosides, saponins, flavonoids, and anthraquinones, which can provide hints for the further development of HXJTY into modern preparation to achieve the purpose of taking less dosage and strong efficacy. In addition, the result in Figure 3 showed that there was a significant difference of the contents of each component in four batches of HXJTY. Therefore, to ensure the efficacy and consistency of therapeutic effect, the content of quality markers should be monitored.

The biofunctional analysis of GO showed that the potential target genes of HXJTY for treating T2DM were closely related to peptidase activity, hydrolase activity, phosphatase activity, and cofactor binding. This suggests that HXJTY for treating T2DM may regulate multiple complex biological processes. It can be seen from KEGG pathway enrichment analysis that 125 common target genes were mainly distributed to PI3K-Akt, MAPK, AGE-RAGE, Rap1, and other multiple signaling pathways. Previous studies have also shown that PI3K-Akt [25], MAPK [26], AGE-RAGE [27], and Rap1 [28] were important signaling pathways for the treatment of T2DM. Among them, PI3K-Akt signaling pathway has been reported to be involved in regulating many life phenomena such as cell proliferation, differentiation, growth, and apoptosis and play crucial roles in the pathogenesis of T2DM, obesity, and inflammation [25]. Numerous studies have found that AGE-RAGE signaling pathway not only mediated the damage of many kinds of cells but also contributed to the endothelial dysfunction in coronary arterioles [27]. Kay et al. also reported that the AGE-RAGE signaling heavily influenced both cellular and systemic responses to increase bone matrix proteins through p38 MAPK, NFκB, ERK1/2, TGF-*β*, fetuin-A, and PKC signaling pathways in both calcification and hyperglycemic conditions [29].

In conclusion, combining HPLC-MS mass spectrometry analysis with network pharmacology research method, the quality markers and mechanism of HXJTY for treating T2DM were revealed. Our findings could provide a useful approach to control the quality of HXJTY as to assure the safety and efficacy of HXJTY in clinical application. According to the predicted results, network pharmacology suggests a possible research orientation for the study of drug mechanism, and it has a potential application value for mechanism research of TCM formulation.

## Figures and Tables

**Table 1:** Information for quantitative analysis from the monitoring ion.

No.	Component	Rt (min)	Ionization mode	m/z	Herb	Compound class
1	Catalpol	5.06	[M + HCOO]^−^	407.10	RR^a^	Glycosides
2	Aucubin	8.40	[M + HCOO]^−^	391.10	RR	Glycosides
3	Gallic acid	8.58	[M − H]^−^	169.10	RR, RRER^b^	Organic acid
4	Rhmannioside D	9.67	[M + HCOO]^−^	731.10	RR	Glycosides
5	Leonuride	14.18	[M + HCOO]^−^	393.10	RR	Glycosides
6	Hydroxysafflor yellow A	18.12	[M − H]^−^	611.10	CF^c^	Flavonoids
7	Amygdalin	18.78	[M + HCOO]^−^	502.10	PS^d^	Glycosides
8	Echinacoside	19.87	[M − H]^−^	785.15	RR	Glycosides
9	Calycosin-7-*O*-*β*-D-glucoside	22.24	[M + H]^+^	447.05	AR^e^	Flavonoids
10	Acteoside	22.53	[M − H]^−^	623.15	RR	Glycosides
11	Ononin	26.59	[M + HCOO]^−^	475.05	AR	Glycosides
12	Calycosin	29.34	[M + H]^+^	285.10	AR	Flavonoids
13	Astragaloside IV	31.77	[M + HCOO]^−^	829.35	AR	Saponins
14	Astragaloside III	32.35	[M + HCOO]^−^	829.35	AR	Saponins
15	Ophiopojaponin C	33.19	[M + HCOO]^−^	931.35	OR^f^	Saponins
16	Astragaloside II	33.93	[M + HCOO]^−^	871.40	AR	Saponins
17	Formononetin	34.99	[M + H]^+^	269.10	AR	Flavonoids
18	Isoastragaloside II	35.25	[M + HCOO]^−^	871.40	AR	Saponins
19	Rhein	37.61	[M − H]^−^	283.05	RRER	Anthraquinones
20	Astragaloside I	38.12	[M + HCOO]^−^	913.35	AR	Saponins
21	Isoastragaloside I	39.33	[M + HCOO]^−^	913.35	AR	Saponins
22	Emodin	43.27	[M − H]^−^	269.05	RRER	Anthraquinones

^a^RR: Rehmanniae Radix; ^b^RRER: Rhei Radix et Rhizoma; ^c^CF: Carthami Flos; ^d^PS: Persicae Semen; ^e^AR: Astragali Radix; ^f^OR: Ophiopogonis Radix.

**Table 2:** Result of pathway enrichment (top 20).

ID	Description	*P* value	Target gene count
hsa04151	PI3K-Akt signaling pathway	3.19 × 10^−6^	17
hsa04010	MAPK signaling pathway	1.12 × 10^−6^	16
hsa04933	AGE-RAGE signaling pathway	1.04 × 10^−11^	15
hsa04015	Rap1 signaling pathway	1.06 × 10^−5^	13
hsa04915	Estrogen signaling pathway	6.86 × 10^−7^	12
hsa04014	Ras signaling pathway	1.36 × 10^−4^	12
hsa04625	C-type lectin receptor signaling pathway	2.84 × 10^−7^	11
hsa04066	HIF-1 signaling pathway	4.59 × 10^−7^	10
hsa04926	Relaxin signaling pathway	2.49 × 10^−6^	11
hsa04068	FoxO signaling pathway	2.90 × 10^−6^	11
hsa04022	cGMP-PKG signaling pathway	2.97 × 10^−6^	10
hsa04668	TNF signaling pathway	4.83 × 10^−6^	10
hsa04152	AMPK signaling pathway	8.98 × 10^−6^	10
hsa04910	Insulin signaling pathway	2.88 × 10^−5^	10
hsa04072	Phospholipase D signaling pathway	5.60 × 10^−5^	10
hsa04150	mTOR signaling pathway	7.42 × 10^−5^	10
hsa04920	Adipocytokine signaling pathway	6.00 × 10^−7^	9
hsa04211	Longevity regulating pathway	5.27 × 10^−6^	9
hsa04657	IL-17 signaling pathway	8.30 × 10^−6^	9
hsa04620	Toll-like receptor signaling pathway	1.90 × 10^−5^	9

**Table 3:** Interaction network results of 21 active components.

Component	Degree	Target gene
Catalpol	1	LGALS3
Aucubin	1	LGALS3
Gallic acid	27	CA1, TTR, IGF1R, SERPINE1, COMT, ALB, KDR, CNR2, ESR1, PARP1, FASN, ELANE, ADRA2A, ADRA2C, ADRA2B、ADRB2、DRD3、PTPN1、PTGS2, EGFR, DPP4, CNR1, FTO, OPRM1, PTGS1, RXRG, RXRA
Rhmannioside D	17	CA1, FGF2, HRAS, FN1, F2, F7, SIRT1, AKR1B1, PTPN1, SLC5A2, ACE, SELP, MMP2, MMP12, STAT3, PRKCD, RORC
Leonuride	1	SLCO1B3
Hydroxysafflor yellow A	24	CA1, PTPN1, SLC6A4, PTGS2, PRKCD, TERT, IKBKB, GSTM1, ACP1, PTPN2, GSK3B, AKR1B1, ABCB1, MMP2, MMP12, HSD11B1, APP, CNR1, MTOR, HMGCR, TLR9, F2, F10, IL1B
Amygdalin	1	LGALS3
Echinacoside	2	ALDH2, LGALS3
Calycosin-7-*O*-*β*-D-glucoside	31	TNF, ALDH2, AKR1B1, CA1, XDH, HRAS, MAPK14, F10, ADRA2C, PARP1, ABCB1, HCAR2, PTGS1, ADRA2A, EGFR, CD38, MIF, MMP9, MMP2, MMP8, ALB, MMP12, PTPN22, TLR9, PPARA, APP、LGALS3, IRAK4, SLC5A2, CASP1, INSR
Ononin	3	ALDH2, MTTP, SHBG
Calycosin	27	ABCB1, MIF, PPARA, ESR1, TLR9, EGFR, PTGS1, ALDH2, XDH, CA1, PTPN1, PON1, PLAT, F10, TNF, IGFBP3, AKR1B1, SNCA, GCGR, ADRB2, KDM1A, TNNC1, TNNT2, TNNI3, IGFBP5, IGFBP2, IGFBP1, PPARG, ALOX5
Astragaloside IV	31	VEGFA, FGF1, FGF2, HPSE, LGALS3, ADRA2A, ABCB1, ADRA2C, ADRA2B, STAT3, AKT2, AKT1, CNR1, CNR2, FAAH, ADRB2, MTOR, PIK3CG, RORC, F10, IGF2R, DRD3、F7, VDR, PIK3CA, PIK3R1, PTPN2, EGFR, MMP1, SLC6A4, SELL, SELP
Astragaloside III	26	VEGFA, FGF1, FGF2, HPSE, LGALS3, RORC, ADRA2A, ADRA2C, ADRA2B, DRD3、STAT3、VDR, SLC5A1, CNR1, CNR2、HSD11B2、HSD11B1, ABCB1, ADRB2, MMP2, MMP12, IGF2R, GLB1, PPARG, ADRB3, IGF1R
Ophiopojaponin C	28	STAT3, F2, HSD11B2, HSD11B1, PTPN1, ADRB2, MMP2, MMP8, SLC5A2, SLC5A1, AKR1B1, ADRA2A, ADRA2C, ADRA2B, OPRM1, IGF1R, CASP1, DGAT1, EPHX2, LIPC, KCNH2, PRKCD, NR3C1, MMP1, FAAH, ABCG2, DPP4, ADRB3
Astragaloside II	31	VEGFA, FGF1, FGF2, HPSE, ABCB1, ADRA2A, ADRA2C, ADRA2B, STAT3, LGALS3, VDR, RORC, REN, IKBKB, ADRB2, SLC5A1, CNR1, CNR2, DRD3, EGFR, FAAH, IGF1R, IGF2R, AKT2, AKT1, INSR, FKBP5, LIPC, MMP1, TLR9, MTOR
Formononetin	4	ESR1, MTTP, AKT1, SHBG
Isoastragaloside II	32	VEGFA, FGF1, FGF2, HPSE, ABCB1, ADRA2A, ADRA2C, ADRA2B, STAT3, LGALS3, VDR, RORC, REN, IKBKB, ADRB2, SLC5A1, CNR1, CNR2, DRD3, EGFR, FAAH, IGF1R, IGF2R, AKT2, AKT1, INSR, FKBP5, LIPC, MMP1, TLR9, MTOR, SLCO1B3
Rhein	11	ALOX5, MAPK8IP1, CYP1A2, CYP2C9, CYP3A4, CYP3A43, CYP3A5, GSTP1, NR1H2, PTGS1, PTGS2
Astragaloside I	27	VEGFA, FGF1, FGF2, RORC, ADRA2A, ADRA2C, ADRA2B, VDR, LGALS3, MTOR, HPSE, MAPK14, HLA-A, P2RY12, SLC5A1, REN, ABCB1, DRD3, LIPC, MMP9, MMP8, IKBKB, PPARG, F3, F7, CTSD, MMP3, MMP1
Isoastragaloside I	28	VEGFA, FGF1, FGF2, HPSE, ADRA2C, ADRA2B, ABCB1, VDR, LGALS3, DRD3, RORC, ADRA2A, CNR1, CNR2, SLC5A1, HLA-A, LIPC, TERT, ALDH2, PRKCD, EDNRB, IGF1R, DPP4, REN, MTOR, MMP9, MMP8, AKT1
Emodin	8	ALOX5, CYP1A2, CYP2C9, CYP3A4, CYP3A43, CYP3A5, GSTP1, NR1H2

## Data Availability

The data used to support the findings of this study are available from the corresponding author upon request.

## Abbreviations

HXJTY: Huo-Xue-Jiang-Tang Yin
T2DM: Type 2 diabetes mellitus
TCM: Traditional Chinese medicine
AR: Astragali Radix
RR: Rehmanniae Radix
CF: Carthami Flos
OR: Ophiopogonis Radix
RRER: Rhei Radix et Rhizoma
PS: Persicae Semen
DR: Dioscoreae Rhizoma
PR: Pseudostellariae Radix
TCMIP: Integrative Pharmacology-based Research Platform of Traditional Chinese Medicine
PPI: Protein-protein interaction
GO: Gene ontology
KEGG: Kyoto Encyclopedia of Genes and Genomes.
